# Analysis of oral microbiome from fossil human remains revealed the significant differences in virulence factors of modern and ancient *Tannerella forsythia*

**DOI:** 10.1186/s12864-020-06810-9

**Published:** 2020-06-15

**Authors:** Anna Philips, Ireneusz Stolarek, Luiza Handschuh, Katarzyna Nowis, Anna Juras, Dawid Trzciński, Wioletta Nowaczewska, Anna Wrzesińska, Jan Potempa, Marek Figlerowicz

**Affiliations:** 1 Institute of Bioorganic Chemistry, Polish Academy of Sciences, 61-704 Poznan, Poland; 2 Department of Human Evolutionary Biology, Institute of Anthropology, Faculty of Biology, Adam Mickiewicz University in Poznan, 61-614 Poznan, Poland; 3 Department of Human Biology, Faculty of Biological Sciences, Wroclaw University, 50-138 Wroclaw, Poland; 4 Anthropological Laboratory, Museum of the First Piasts at Lednica, 62-261 Lednogora, Poland; 5 Faculty of Biochemistry, Biophysics and Biotechnology, Jagiellonian University, 30-387 Krakow, Poland; 6 Department of Oral Immunity and Infectious Diseases, University of Louisville School of Dentistry, Louisville, KY 40202 USA; 7 Institute of Computing Science, Poznan University of Technology, 60-965 Poznan, Poland

**Keywords:** aDNA, Ancient genomics, *T. forsythia*, Oral microbiome, Comparative genomics

## Abstract

**Background:**

Recent advances in the next-generation sequencing (NGS) allowed the metagenomic analyses of DNA from many different environments and sources, including thousands of years old skeletal remains. It has been shown that most of the DNA extracted from ancient samples is microbial. There are several reports demonstrating that the considerable fraction of extracted DNA belonged to the bacteria accompanying the studied individuals before their death.

**Results:**

In this study we scanned 344 microbiomes from 1000- and 2000- year-old human teeth. The datasets originated from our previous studies on human ancient DNA (aDNA) and on microbial DNA accompanying human remains. We previously noticed that in many samples infection-related species have been identified, among them *Tannerella forsythia*, one of the most prevalent oral human pathogens. Samples containing sufficient amount of *T. forsythia* aDNA for a complete genome assembly were selected for thorough analyses. We confirmed that the *T. forsythia*-containing samples have higher amounts of the periodontitis-associated species than the control samples. Despites, other pathogens-derived aDNA was found in the tested samples it was too fragmented and damaged to allow any reasonable reconstruction of these bacteria genomes. The anthropological examination of ancient skulls from which the *T. forsythia*-containing samples were obtained revealed the pathogenic alveolar bone loss in tooth areas characteristic for advanced periodontitis. Finally, we analyzed the genetic material of ancient *T. forsythia* strains. As a result, we assembled four ancient *T. forsythia* genomes - one 2000- and three 1000- year-old. Their comparison with contemporary *T. forsythia* genomes revealed a lower genetic diversity within the four ancient strains than within contemporary strains*.* We also investigated the genes of *T. forsythia* virulence factors and found that several of them (KLIKK protease and *bspA* genes) differ significantly between ancient and modern bacteria.

**Conclusions:**

In summary, we showed that NGS screening of the ancient human microbiome is a valid approach for the identification of disease-associated microbes. Following this protocol, we provided a new set of information on the emergence, evolution and virulence factors of *T. forsythia,* the member of the oral dysbiotic microbiome.

## Background

Currently, periodontitis is a common condition that affects approximately 15–20% of the worldwide population (age 35–44 years; WHO. 2012. Fact sheet N318: oral health. WHO, Geneva, Switzerland). This prevalence correlates well with the prevalence of periodontitis in adults (aged > 30 years) in the United States, at 46%, with 8.9% having severe disease [1]. The molecular pathogenicity of periodontitis as a microbiota-shift disease is still far from fully understood [2]. According to a well-accepted paradigm, the disease is driven by dysbiotic bacterial flora composed of the red complex oral bacteria (*Porphyromonas gingivalis*, *Treponema denticola* and *T. forsythia*) as well as a cohort of newly recognized periodontal pathogens [3]. In a subgingival biofilm, they form a tightly knit community engaged in competitive and cooperative interactions [4]. A futile attempt of the host to eradicate dysbiotic biofilm fuels a chronic inflammatory reaction in the infected periodontium. In genetically susceptible hosts, this inflammation leads to dissolution of the periodontal ligament, alveolar bone resorption, deep periodontal pocket formation and eventual tooth loss [5].

Among recognized pathogens, *T. forsythia* is grossly under investigated, and only a handful of its virulence factors have been characterized to date [6]. This lack of knowledge is perplexing in light of a growing body of evidence that *T. forsythia* is strongly associated with periodontitis and must largely contribute to the pathogenicity of the microbiota in subgingival plaque [4, 7, 8]. To date, several virulence factors of *T. forsythia* have been reported [6]. The list of them is still growing and includes: (i) proteases (KLIKK, PrtH) [9, 10] that protect the bacterium from being killed by complement and bactericidal peptides [11–13]; (ii) dipeptidyl peptidase IV (DppIV) that is implicated in host tissue destruction [14, 15]; (iii) miropin that acts as a bacterial inhibitor of host broad-range proteases, some of them contributing to antibacterial activity of the inflammatory milieu [16]; (iv) glycosidases (SusB, SiaHI, NanH, and HexA) that degrade oligosaccharides and proteoglycans in saliva, gingival and periodontal tissues and promote disease progression [17–20]; and (v) the OxyR protein responsible for biofilm activity that facilitates and/or prolongs bacterial survival in diverse environmental niches [21].

Alike *P. gingivalis, T. forsythia* uses a type IX secretion system (T9SS) composed of PorK, PorT, PorU, Sov and several other conserved proteins to deliver virulence factors to the bacterial surface [22]. The T9SS cargo includes KLIKK proteases, BspA protein and components of the semi-crystalline S-layer (TfsA and TfsB). The latter provides bacteria with a protective shielding and promotes microbe adhesion [23, 24]. In addition, these proteins are heavily glycosylated with a unique complex O-linked decasaccharide containing nonulosonic acids, either legionaminic acid (Leg) or pseudaminic acid (Pse), a sialic acid-like sugars implicated in evasion of the host immune response. Of note, the occurrence of Leg or Pse is strain-specific {Bloch, 2019 #649}. Among the surface-anchored proteins, BspA is currently the best characterized *T. forsythia* virulence factor. BspA was shown to be involved in binding to fibronectin and fibrinogen [25]; to mediate interactions with other bacteria (among others with *T. denticola* [26]) and to induce bone loss in mice [26]. BspA belongs to the family of leucine-rich repeat (LRR) proteins. It is composed of 20 tandem LRR domains in the N-terminal region and 4 immunoglobulin-like (Ig-like) domains typically found in bacteria. The LRR region plays a role in protein-protein interactions. The function of BspA Ig-like domains has not yet been determined, but it is suggested that they may stabilize the tertiary structure of LRRs [6]. Sequencing of the *T. forsythia* ATCC 43037 genome, apart from *bspA* (*BFO_RS14480*), revealed five more genes (*BFO_RS14345* (*bspB*), *BFO_RS08355*, *BFO_RS14330*, *BFO_RS14330*, and *BFO_RS14330*) encoding putative BspA-like proteins. Among them, BspB requires special attention because, in contrast to other BspA-like proteins, it possesses both LRR and Ig-like domains. While the amino acid sequence of the LRR region of BspA and BspB is different to a large extent, the Ig-like regions displayed 99% amino acid sequence similarity. The BspB protein was identified in the *T. forsythia* outer membrane proteome, but its function is still unknown [27].

In earlier studies, we identified a wide spectrum of bacterial species in 1000- and 2000- year-old human remains and showed that some of them most likely accompanied their hosts before their deaths [28]. Here, we analyze the compositions of the ancient oral microbiomes. We used *T. forsythia* presence as a marker for the potential occurrence of periodontitis and showed statistically significant differences in the amount of DNA of periodontitis-associated bacteria in samples with the highest amounts of *T. forsythia* ancient DNA (aDNA) comparing to the reference samples. We also attempted a complete genome assembly of *T. forsythia* as three samples contained sufficient amounts of aDNA derived from this bacterium. Subsequently, we investigated the evolution of the *T. forsythia* genome, particularly focusing on genes encoding bacterial virulence factors that contribute to periodontitis development. To date, little is known about the genetic diversity of this common pathogen, especially in the context of its infection-associated genes. Comparative studies of whole *T. forsythia* ancient and modern genomes, which we performed for the first time, shed light on this matter, revealing huge sequence variability in some virulence-related genes. Moreover, the Roman Iron Age and medieval genomes analyses brought important information on the origin and evolution of this bacterium through ages and, most importantly, on the evolutionary conservation of its virulence factors.

## Results

While examining 1000–2000-year old human skulls we found that some of them had typical for periodontitis pathogenic alveolar bone lesions in tooth areas. This observation roused the question whether it is possible to determine the nature of these lesions by identifying DNA biomarkers of periodontal infection in the oral microbiome collected from the ancient human remains. To this end, we scanned next-generation sequencing (NGS) metagenomic datasets obtained for 344 human skeletal remains by mapping reads to the database of unique clade-specific marker sequences [29]. The metagenomic dataset was generated with DNA extracted from human teeth dated from the 1st to the sixteenth c. AD. The biological material came from archaeological sites distributed across Poland.

An analysis of NGS data generated separately for each of the 344 studied individuals allowed us to estimate that nine samples contained more than 3% of *T. forsythia* DNA (Supplementary Table 1, [29]). The NGS datasets obtained for these nine samples were mapped against the *T. forsythia* reference genome (NC_016610.1), showing that for three out of nine samples, the average nucleotide coverage was > 5, and for more than 80% of the *T. forsythia* genome, the nucleotide coverage was ≥3 (Supplementary Figure 1 A). These three samples were selected for further analyses. Additionally, the studied group was extended with the published data on the teeth microbiome of an individual living in Dalheim, Germany in the 10th–twelfth c. AD in which sample *T. forsythia* was also identified [30].

### Identification of periodontitis-associated bacteria in ancient samples

Anthropological analyses revealed that all four skulls from which *T. forsythia* DNA was isolated had characteristic for periodontitis lesions in the tooth area (see Supplementary Material “Anthropological description of analyzed individuals” and Supplementary Figure 2 for PCA0088, PCA0198, and PCA0332 and [30] for G12). Accordingly, we investigated the overall microbial content of these DNA samples to determine the presence of other bacterial species that have been shown to be associated with periodontitis in humans [31]. Metagenomic analysis involving MetaPhlAn2 [29] revealed that bacteria consisted of 82.05, 99.57, 99.44, 98.49% of the PCA0088, PCA0198, PCA0332 and G12 samples, respectively. The microbial content of each sample is shown in Supplementary Figure 7. Overall, 1 archaeal and 21 bacterial classes were identified. In the PCA0088 and PCA0332 samples, *Clostridia* was the most abundant class, consisting of 35.50 and 25.43% of all bacteria, respectively. In the PCA0198 sample, *Actinobacteria* was the majority, consisting of 28.74% of all bacteria; in G12, *Bacilli* constituted 27.99% of the bacterial component. The *Bacteroidetes* class, to which *T. forsythia* belongs*,* comprised 3.06, 4.49, 3.97, and 3.02% of all bacteria in PCA0088, PCA0198, PCA0332, and G12, respectively. The characteristics of the genera identified within all of the classes uncovered the prevalence of taxa typical of human flora (85.09, 96.23, 97.48, and 96.05% in PCA0088, PCA0198, PCA0332, and G12), including 75.34, 77.63, 90.2, and 81.04% of oral genera, respectively. The remaining genera consisted of ubiquitous environmental taxa typical of a wide range of soils and waters. At the species level, *T. forsythia* accounted for 5.91, 22.76, 4.23 and 2.14% of bacterial species in the samples PCA0088, PCA0198, PCA0332, and G12, respectively, and was the most abundant species in the sample PCA0198. *P. gingivalis* and *T. denticola,* which together with *T. forsythia* constitute the “red complex”, were detected in all four ancient samples. *P. gingivalis* represented 0.45, 3.62, 1.07, and 0.35% of bacteria, and *T. denticola* represented 1.52, 2.95, 3.31, and 0.78% of bacteria in PCA0088, PCA0198, PCA0332, and G12, respectively. Additionally, we discovered in at least one of the four samples the following periodontitis-associated species: *Filifactor alocis*, *T. medium*, *T. vincentii*, *Lachnospiraceae oral taxon 107*, *P. intermedia* or *G. elegans.*

To check whether the amount of pathogenic species in the four analyzed samples differed from that in other ancient samples, we compared the microbial content of PCA0088, PCA0198, PCA0332, and G12 with the content found in the other 17 ancient samples in which human oral species consisted of > 75% [28] (Supplementary Table 5). The comparison of the content of periodontitis-associated species showed statistically significant differences. In particular, *T. medium* and *T. vincentii* abundances revealed the highest statistical significance (t-test, *p*-val < 0.0001) followed by *P. intermedia* and *P. gingivalis* abundances (t-test, *p*-val < 0.01) and by *T. forsythia* and *G. elegans* abundances (t-test, *p*-val < 0.05). It must be pointed out that there was no anthropological information on the inflammatory lesions on the 17 skeleton jaws from which the aDNA samples served in this analysis as references. Therefore, it is likely that the reference set contained samples derived from periodontitis sites. If so, this will only strengthen the significant difference in composition and abundance of periodontopathogenic species in the analyzed sets of samples.

### Assembly of the ancient *T. forsythia* genomes

**Fig. 1:**
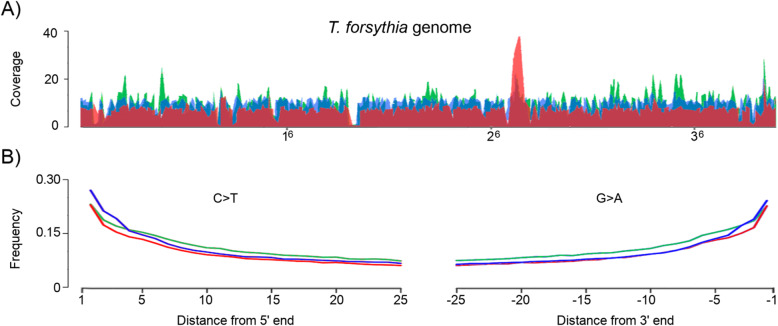
**a** The coverage plot of reads mapped to the 3.4 Mb large *T. forsythia* reference genome (NC_016610.1). The plot shows the average coverage calculated for 400 windows. **b** aDNA damage pattern. Plots of the C > T and G > A nucleotide transition frequencies at the 5′ and 3′ ends of DNA fragments, respectively. Red: PCA0088, blue: PCA0198, green: PCA0332

Based on NGS data obtained for the samples selected for the study (samples with the high levels of *T. forsythia* aDNA, we were able to assemble four variants of the full-length *T. forsythia* genome. Each variant was named after the sample IDs from which aDNA was isolated: PCA0088, PCA0198, and PCA0332. Sample PCA0088 was obtained from an individual buried in Masłomęcz during the Roman Iron Age (2nd-fourth c. AD) [32, 33], sample PCA0198 was from an individual living in Ląd during the Early Medieval Age (10th–twelfth c. AD, Supplementary Figure 3) [34], sample PCA0332 was from an individual living in Ostrów Lednicki during the Medieval Age (12th–thirteenth c. AD) and sample G12 was from an individual living in Dalheim, Germany in the 10th–twelfth c. AD [30]. Overall, 321,886, 444,721, 427,421, and 325,568 unambiguous reads from the PCA0088, PCA0198, PCA0332, and G12 samples, respectively, were mapped to the reference genome sequence (NC_016610.1), with average coverage of 7.03, 9.81, 9.71, and 6.95, respectively (Fig. 1a, Supplementary Table 1). To verify whether the assembled *T. forsythia* genomes were of ancient origin and whether the bacteria accompanied the humans before death, we analyzed the signatures of age-related DNA damage. aDNA damage patterns were evaluated using mapDamage2.0, which simulates the posterior distribution of deamination in DNA [35]. The analysis of reads mapped to the *T. forsythia* reference genome revealed typical aDNA damage patterns, as presented in Fig. 1b. An increase of C > T (and G > A) nucleotide transition frequencies up to 25% at the 5′ (and 3′) end of DNA fragments was observed. Sample G12 was not included in this analysis because the polymerase that was used for library preparation (Phusion Finnzymes [30];) is not able to replicate through uracil; thus, age-related DNA modifications could not be assessed [36]. The length distribution of mapped reads (Supplementary Figure 1 B) showed that the *T. forsythia* average aDNA fragment was 82, 85 and 88 nt long in PCA0088, PCA0198, and PCA0332, respectively. As a single-end approach was applied for G12 sequencing, which does not allow read merging, the average read length of this sample (73 nt) could not be directly compared with the read lengths of pair-end libraries used in our study. In summary, the read length distribution and DNA damage patterns supported the ancient origin of the sequenced *T. forsythia* DNA.

### Comparative analysis of the reconstructed and modern *T. forsythia* genomes

To investigate contemporary and ancient *T. forsythia* genome diversity, we extended the studied group of four ancient genomes with ten publicly available modern genomes of *T. forsythia* (Table 1). First, we analyzed single nucleotide polymorphisms (SNPs, GATK tools [37]) and created an SNP-based phylogenetic tree with FastTree [38]. Second, we determined the deletion distribution.

**Table 1:** The list of contemporary *T. forsythia* strains with sequenced genomes

Strain	NCBI BioProject id	Source	Location	Assembly	SNPs
92A2 - the reference	PRJNA319	Human periodontal pocket	USA, Massachusetts	Complete genome	N/A
3313	PRJDB1007	Human oral cavity	Japan, Tokyo	Complete genome	26,310
KS16	PRJDB1008	Human oral cavity	Japan, Tokyo	Complete genome	25,880
NSLJ	PRJNA401301	Human Subgingival plaque	UK, London	Contig	24,171
NSLK	PRJNA401301	Human Subgingival plaque	UK, London	Contig	25,744
ATCC 43037	PRJNA548889	Human periodontal pocket	USA, N/K	Scaffold	26,806
UB20	PRJEB15383	Human Subgingival plaque	USA, New York	Scaffold	24,845
UB22	PRJEB15383	Human Subgingival plaque	USA, New York	Scaffold	21,796
UB4	PRJEB15383	Human Subgingival plaque	USA, New York	Scaffold	27,183
9610	PRJNA340021	Human periodontal pocket	USA, Washington	Scaffold	24,232

The analysis involving the *T. forsythia* reference genome (92A2) showed that Roman Iron Age *T. forsythia* PCA0088 had 1167 SNPs, while in the medieval *T. forsythia* PCA0198, PCA0332 and G12 genomes, we identified 2645, 1933 and 1374 SNPs, respectively. For each modern *T. forsythia* genome, an average of ~ 25,000 SNPs were identified. The relatively low number of SNPs identified in the ancient strains can be explained by incompleteness of the genomes, age-related aDNA modifications, restricted criteria of SNP calling as well as by their location on the SNP tree (see below). Generally, most SNPs were identified within known genes (92.58%), including 1.33% of SNPs in virulence-associated genes. That yields, on average, 21 SNPs per gene and 28 SNPs per virulence factor gene (Supplementary Table 2 A).

**Fig. 2:**
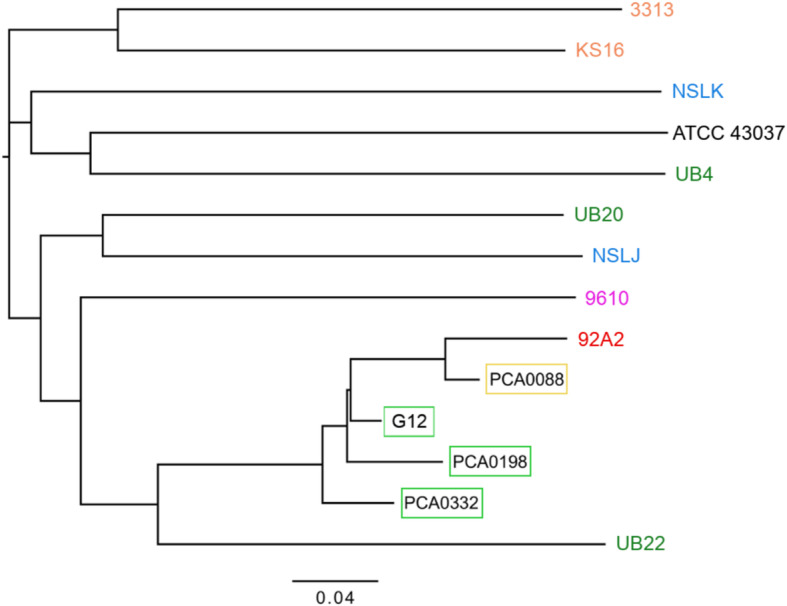
The SNP phylogenetic tree showing the position of the Roman Iron Age (PCA0088) and medieval (PCA0198, PCA0332, and G12) *T. forsythia* genomes with respect to the genomes of modern *T. forsythia* identified worldwide

A SNP-based phylogenetic tree of *T. forsythia* contemporary genomes was constructed based on 64,413 SNPs that were discovered in at least one of the analyzed genomes, and the reference nucleotide was determined for all of the remaining modern genomes (Supplementary Table 2 A). Two Japanese genomes (KS16 and 3313) grouped together on the phylogenetic tree; however, the positions of genomes isolated in London (NSLK and NSLJ) and those obtained in the same location in the USA (UB4, UB20, and UB22) did not correlate with their geographical regions. Ancient *T. forsythia* genomes were subsequently “projected” (with pplacer [39]) on the previously generated tree of modern genomes (Fig. 2). We did not include ancient genomes during the construction of the initial phylogenetic tree, as the number of SNPs determined for the four ancient genomes was ~ 10-fold lower than the average number of SNPs identified in the contemporary genomes. This approach ensured that the similarity of ancient and contemporary *T. forsythia* was not caused by the reduced number of identified SNPs. All four ancient genomes were placed within the 92A2, 9610 and UB22 cluster. The oldest *T. forsythia* genome, PCA0088, which is geographically distinct from the three medieval genomes, displayed the closest relationship with 92A2. Further, to confirm the placement of ancient *T. forsythia* on the SNP phylogenetic tree, we repeated the analysis, but with more restricted criteria of nucleotide calling in the ancient genomes. To call a SNP/reference nucleotide, at least 10-fold coverage was required (instead of the initially used 3-fold coverage, Supplementary Figure 4 A). Despite the use of more restrictive criteria, the general location of the four ancient genomes in the phylogenetic tree was the same. They remained within the 92A2, 9610 and UB22 cluster, though PCA0198 was placed closest to 92A2, whereas PCA0088 and G12 were the most distant. These differences, however, might be caused by the reduced number of SNPs, which was especially meaningful to the two ancient genomes with the lowest genome coverage (PCA0088 and G12). In the third attempt to construct the phylogenetic tree, we used a threshold of min. 3-fold coverage, and we also excluded SNPs identified in reads < 70 nt long (Supplementary Figure 4 B) because Green et al. [40] showed that shorter reads containing a SNP are more likely to be unmapped because they carry less information to place them uniquely in the genome. In the next attempt, we excluded all C/T and G/A SNPs identified in the PCA0088, PCA0198, PCA0332, and G12 genomes, as transitions C > T and G > A caused by DNA damage could be misidentified as original SNPs (Supplementary Figure 4 C). The ancient genomes again positioned closest to the 92A2 genome and arranged in the same way in both SNP trees (Supplementary Figure 4 B, C). In comparison to their locations in the original SNP tree (Fig. 2), the location of the G12 and PCA0198 genomes swapped. This effect might be caused by the fact that G12 had the shortest average read length, making more reads excluded in this sample (Supplementary Figure 4 B), and by the previously mentioned properties of the polymerase used for G12 sequencing, which might lead to C/T and G/A SNP misidentification (Supplementary Figure 4 C). Moreover, the generation of SNP-based phylogenetic trees using 10 contemporary genomes and one ancient genome, again confirmed the latter one is always located next to the 92A2 genome (Supplementary Figure 4 D-G). Lastly, we repeated the computations using ATCC 43037 genome [41] as a reference. We identified 6541, 6360, 4711, 6635 SNPs for PCA0088, PCA0198, PCA0332 and G12 respectively (Supplementary Table 2 B). That is ~ 4–5 fold more than when using 92A2 as a reference. This result is an obvious consequence of the phylogenetic relations among the studied *T. forsythia* isolates. Regardless of which genome was applied as a reference (92A2 or ATCC 43037) the ancient ones clustered with 92A2 and 9610 genomes (Supplementary Figure 4 H).

**Fig. 3:**
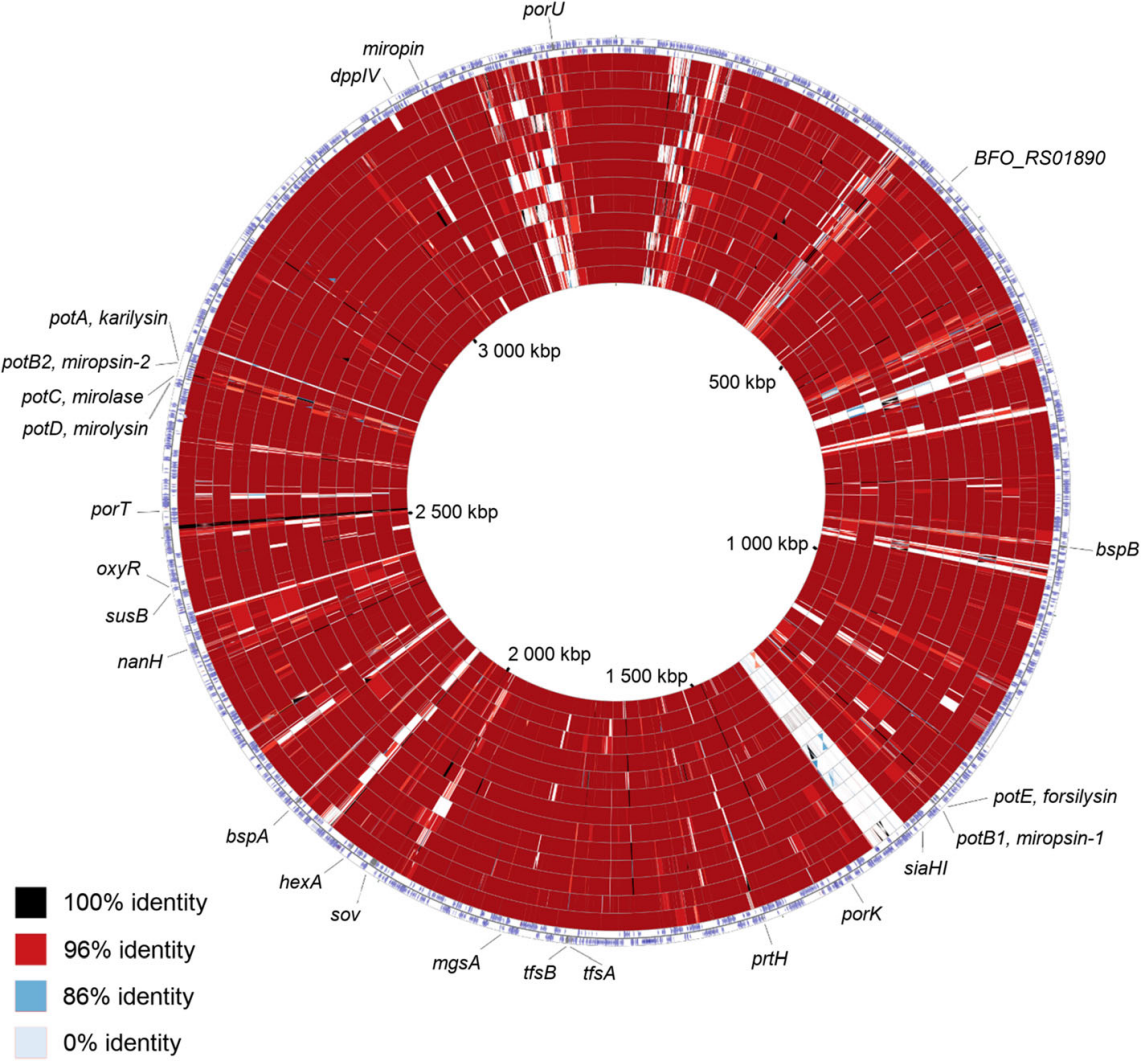
DNA sequence comparison of the *T. forsythia* reference genome to the ancient *T. forsythia* genomes and to publicly available modern *T. forsythia* genomes. The two outermost rings depict the forward and reverse coding strands of the reference genome. The next 13 rings moving towards the inner part of the figure display regions of sequence similarity detected by BLAST comparison between the DNA of the reference genome and the DNA of the 13 compared *T. forsythia* genomes. The following genome order reflects the order of the circles starting from the outer part of the figure and moving towards the inner part: PCA0332, PCA0198, UB20, PCA0088, KS16, UB4, UB22, NSLJ, G12, 3313, NSLK, ATCC 43037, and 9610. Genes associated with *T. forsythia* virulence are labeled in the plot

We also analyzed the sequence identity and deletions in *T. forsythia* genomes using CGView [42]. In comparison to the reference genome, the Roman Iron Age PCA0088 genome had 45 deletions (> 500 nt, Supplementary Table 3), while in the medieval *T. forsythia* PCA0198, PCA0332 and G12 genomes, we identified 44, 54 and 60 deletions (> 500 nt), respectively. As shown in Fig. 3, the deletions occurred within mainly coding regions and were detected both in contemporary and ancient *T. forsythia* genomes; 100, 95.45, 100 and 75% of deletions (> 500 nt) identified in PCA0088, PCA0198, PCA0332 and G12, respectively, were also present in at least one contemporary genome. Additionally, 4.19% of deletions (> 500 nt) were present in all four ancient and in all nine contemporary genomes except for the reference genome. Among them, the largest deletion (45,667 nt) was present in all of the genomes (except the reference genome) and carried tetracycline resistance genes. The high repeatability of deletions in the analyzed genomes is evidence that the absence of most regions in the ancient genomes might not be caused by random DNA degradation.

### *T. forsythia* virulence factors in ancient and contemporary strains

To learn more about changes that occurred in the genetic structure of the *T. forsythia* population within the last 2 thousand years, we assessed variation in the genes that are known to be associated with the pathogenic activity of this bacterium and consequently are crucial for its survival. The analysis also included the *bspB* of unknown function because of its high sequence similarity to the well-known virulence factor, *bspA*. The studied genes are listed in Supplementary Table 4 A, and their genomic location in the 92A2 reference genome is presented in Fig. 3. For KLIKK proteases, we used our in-home sequenced KLIKK *locus* as a reference [10] since it was previously shown to be incorrectly assembled in the 92A2 genome [43]. To evaluate variation in the virulence factor genes, NGS reads used to reconstruct ancient and modern genomes were mapped to the reference genome. As a result, we determined to what extent the virulence genes in the reference genome were covered by NGS reads. If gene coverage was almost complete (> 70% of nucleotides within the region occupied by the gene were covered by NGS reads), we inferred that the gene was present in the analyzed genome. If the gene coverage was moderate, most likely the analyzed genome did not possess this gene or the gene differed remarkably from the one present in the reference genome.

**Fig. 4:**
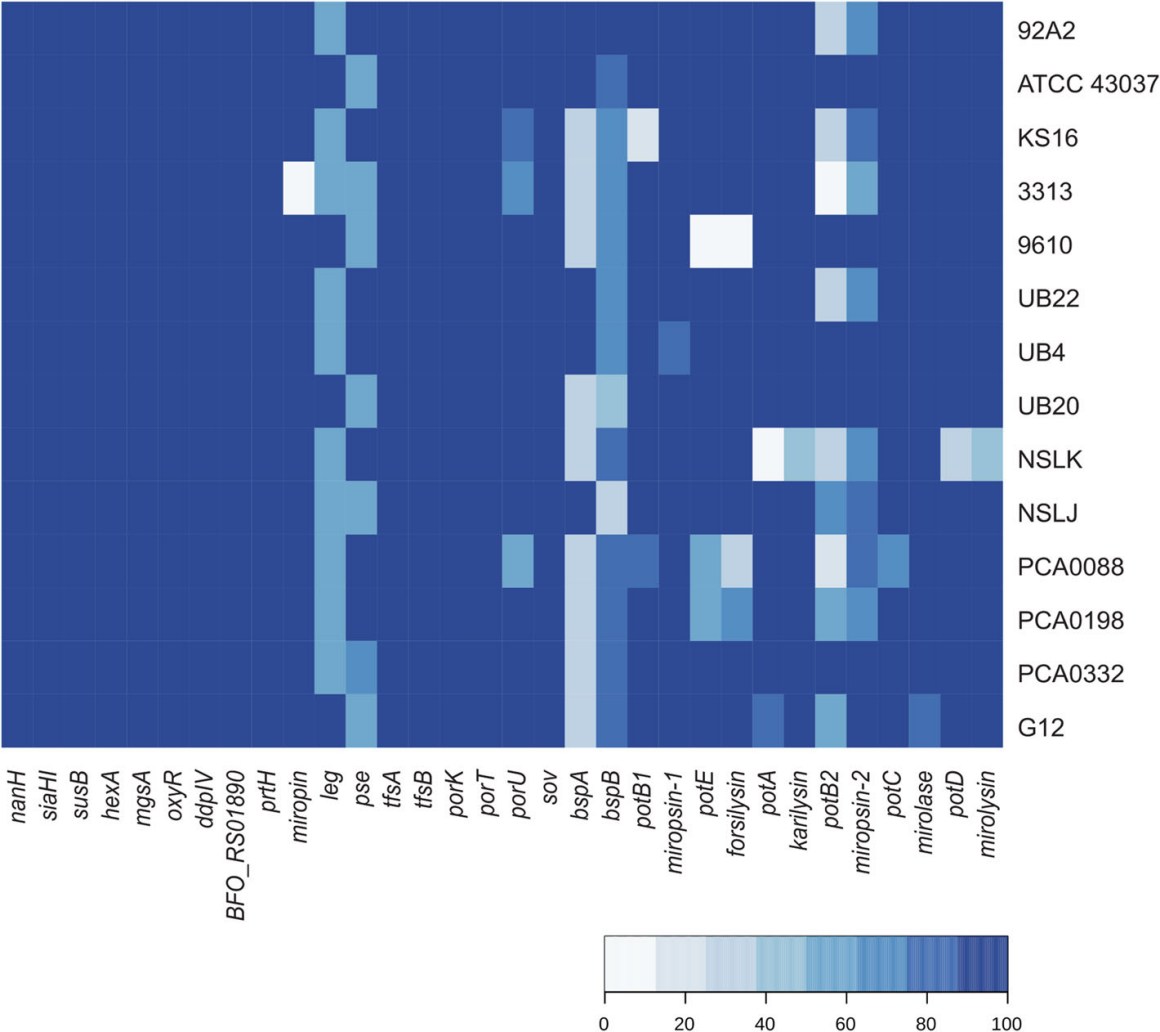
The percentage sequence coverage of modern and ancient known virulence factor genes; the *T. forsythia* 92A2 sequence was used as a reference, apart from *leg* when ATCC 43037 was used as a reference; and KLIKK protease genes when our in-home determined KLIKK protease locus was used as a reference [10]

We found that KLIKK protease genes, together with associated upstream ORFs encoding small lipoproteins (*pot*), were the most variable group among the analyzed virulence factors (Fig. 4, Supplementary Table 4 A, B). Their average gene coverage in ancient genomes was 87.16% (from 17.44% *potB2* in PCA0088 to 100%). The average nucleotide coverage was 4.13 (PCA0088), 7.19 (PCA0198), 9.48 (PCA0332) and 8.33 (G12), which corresponds well to the average nucleotide coverage in the whole analyzed ancient genomes (Supplementary Table 1).

*Miropsin-1, karilysin, mirolase, mirolysin* and accompanying lipoprotein genes (*potB1, potA, potC, potD*) displayed high DNA sequence conservation both in ancient (average gene coverage: 94.64%, from 66.52% in PCA0088 to 100%) and in contemporary genomes (average gene coverage: 93.96%, from 0% in NSLK to 100%, Fig. 4). The only exceptions were the KS16 genome, which seems to be lacking *potB1* (gene coverage: 15.46%) and the NSKL genome, in which gene coverage of *potA* was 0% and *karilysin* was 46.94%, as well as *potD* at 26.85% and *mirolysin* at 46.49%.

*Forsilysin* and accompanying *potE* were well conserved in PCA0332 and G12 (gene coverage: 99.46–100%), but PCA0088 and PCA0198 had only partial coverage of these genes (35.22 and 64.99%, respectively), which implies sequence dissimilarities to those of the reference. Notably, the contemporary 9610 genome seems to lack the two genes, *potE and forsilysin,* as their gene coverage was 10.91 and 11.02%, respectively. Other contemporary strains, however, displayed very high sequence conservation of these two genes (100% gene coverage).

*Miropsin-2* was well covered in all ancient genomes (from 71.38% in PCA0198 to 99% in PCA0332), but the accompanying *potB2* was only partially covered in PCA0088 (17.44%), PCA0198 (52.91%) and G12 (52.13%). Contemporary genomes displayed a similarly variable *potB2* coverage pattern (average gene coverage: 59.60%, from 0 to 100%), which indicated high variability in this gene and even lack of this gene in extreme cases.

It is worth mentioning that in general, we discovered a high correlation between sequence coverage observed for operons composed of KLIKK protease genes directly preceded by genes encoding small lipoproteins referred to as Potempins with inhibitory activity directed specifically against the coexpressed protease (Potempa – data to be published) (Pearson correlation coefficient of 0.80). This finding might suggest a coevolution of those enzymes and their inhibitors.

**Fig. 5:**
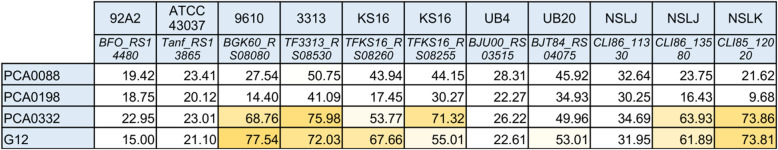
The percentage sequence coverage of the *bspA* in ancient genomes; 11 modern *bspA* sequences were used as a reference (for details, see Methods)

Among other known *T. forsythia* virulence genes, only *bspA* (encoding the bacterial surface-associated protein) was not conserved in the ancient and most contemporary genomes (Fig. 5). Excluding *bspA* and *pot-KLIKK*, in the ancient genomes, the average gene coverage of genes encoding virulence factors was 99.13%. This result implies high evolutionary conservation of virulence-associated genes. The average nucleotide coverage was 7.39 (G12) to 11.88 (PCA0198).

The *bspA* gene was covered in ancient genomes at only 31.3, 29.07, 37.01 and 32.20% (in PCA0088, PCA0198, PCA0332, G12, respectively). Interestingly, within this gene, only the fragment encoding the Ig-like domain was covered (Supplementary Figure 5). Because a highly similar Ig-like coding domain is also present in the *bspB* gene, one can speculate that the observed effect is a result of the mapping procedure rather than a real presence of *bspA* in the ancient genome. The reads mapper most likely would not distinguish between the Ig-like domain sequences present in *bspA* and *bspB*. In accordance with such a presumption, the sequences encoding less conserved LRR domains of *bspA* were not covered in ancient genomes.

In contrast, the analysis of the *bspB* revealed high sequence conservation in the ancient genomes (in both Ig-like and LRR regions). The average *bspB* coverage was 78.93% (from 75.45% in PCA0198 to 81.02% in PCA0088), with the average nucleotide coverage ranging from 7.94 (G12) to 35.56 (PCA0088). Interestingly, the average nucleotide coverage of *bspB* was ~ 5 times higher than the average nucleotide coverage of the PCA0088 genome (7.03) and ~ 3 times higher than the average nucleotide coverage of the PCA0198 genome (9.81) (Supplementary Table 4 A). The above mentioned average *bspB* nucleotide coverage in PCA0088 and in PCA0198, as well as the fact that *bspA* was moderately covered not only in ancient but also in 5 modern genomes (KS16, 3313, 9610, UB20 and NSLK; on average 33.01%, Fig. 5), indicated a general sequence variability of the *bspA* gene. This result inspired us to analyze all *bspA* sequences present in modern *T. forsythia*. Such an analysis would answer the question of whether *bspA* in the ancient genomes is more similar to one of the currently existing forms of *bspA* not present in the reference genome. In total, we identified 11 *bspA* complete sequences listed in Methods, “*bspA* analyses”. Additionally, we identified 47 bspA homologs and included them in the phylogenetic tree (Supplementary Figure 6). The *bspA* was identified in all of the modern genomes (UB22 had an incomplete gene at the contig boundary and was thus excluded from the analysis). Notably, some genomes seemed to have more than one copy of the *bspA*-like gene, particularly KS16 and NSLK.

Subsequently, we mapped reads of ancient NGS datasets to the retrieved 11 modern *bspA* sequences. The results are presented in Fig. 5. The coverage of the *bspA* in samples PCA0088 and PCA0198 did not exceed 50%, regardless of the modern reference used (PCA0088 mapped best to the *bspA* sequence from UB20, 45.92%; PCA0198 to the *bspA* from 3313, 41.09%). PCA0332 achieved 75.98% *bspA* coverage (*bspA* sequence from 3313). Mapping of G12 also resulted in very high *bspA* coverage (max. 77.54% in 9610).

## Discussion

We showed previously that the study of ancient bacterial species in human archaeological samples is possible [28]. In this work, we not only identified *T. forsythia* aDNA in teeth from Roman Iron Age and medieval human skeletons but also attempted complete genome assembly and sequence diversity analyses, paying special attention to virulence-related genes.

The NGS datasets were generated by shotgun sequencing without application of an enrichment procedure, as previously reported for other ancient bacteria [44, 45]. In total, 344 NGS datasets were scanned to trace the presence of *T. forsythia*. Three samples (Roman Iron Age PCA0088 and medieval PCA0198 and PCA0332) contained a sufficient amount of aDNA of this oral pathogen for a genome assembly. Additionally, we included in the analyses previously published NGS data on a *T. forsythia* genome recovered from a medieval skeleton found in Germany [30], as this genome was not previously analyzed in the phylogenetic/virulence gene context. The length distribution of DNA fragments and the damage patterns observed for *T. forsythia* DNA found in the four ancient samples showed damage characteristics typical of aDNA.

Anthropological examination of the four skulls from which aDNA was sampled uncovered alveolar bone loss in each of them, which indicated advanced periodontitis. In keeping with the present-day microbiological etiology of periodontitis, metagenomics analysis showed that in addition to *T. forsythia,* other disease-associated oral bacteria were present in the samples. We identified the “red complex” members (*P. gingivalis* and *T. denticola*) in all four samples. In addition, *F. alocis*, *Lachnospiraceae oral taxon 107*, *T. medium*, *T. vincentii*, *P. intermedia,* and *G. elegans* were found in at least one of the samples. Importantly, the infection-associated species were significantly more abundant in the four selected samples than in 17 ancient reference samples. In particular, statistically significant changes in the amount of bacterial DNA were found for the following species: *T. medium*, *T. vincentii* (t-test, *p*-val < 0.001), *P. intermedia, P. gingivalis* (t-test, *p*-val < 0.01), *T. forsythia,* and *G. elegans* (t-test, *p*-val < 0.05). Taken together, these data showed strong evidence that periodontal problems existed during the Roman Iron Age and medieval times, as they do now, despites different dietary, hygienic and lifestyle habits. The likelihood that the reference set contained samples derived from periodontitis sites only strengthen this general conclusion that the same pathogenic species were responsible for development of periodontitis today and thousands of years ago.

The SNP phylogenetic tree of modern *T. forsythia* fits the epidemiological and geographical contexts, confirming that *T. forsythia* is commonly present in different oral infections worldwide. Only the “Japanese branch” can be easily distinguished (KS16 and 3313). We hypothesize that this might be a consequence of population’s isolation (on the island) or specific dietary habits. The location of other genomes (isolated in Europe and in the USA) on the phylogenetic tree does not always correlate with their geographical origin. This result indicates a worldwide diversity of this oral bacterium. On the other hand, the close location of the ancient genomes on the phylogenetic tree indicates a lower diversity of *T. forsythia* in the Roman Iron Age and medieval central Europe than currently exists.

Analyses of virulence factor genes revealed huge sequence variability in genes encoding the BspA protein and Potempins/KLIKK proteases, as it was shown recently by Zwickl et al. [41]. In all bacterial strains studied except two, 3313 (*miropsin-2*) and NSKL (*karilysin*), KLIKK proteases were directly preceded by ORFs encoding an inhibitor (*potempin*) specific for a downstream protease (Potempa et al., manuscript in preparation). Generally, regarding the *potB2/miropsin* pair, there were more sequence variations in the protease genes than in the accompanying inhibitor genes. Specifically, our findings indicate that *potE, forsilysin*, and *potB2* sequences in PCA0088 and PCA0198, as well as *potB2* in G12, might differ from those present in the modern reference sequences (KP715368 and KP715369). At the same time, *potE* and *forsilysin* were well conserved in all modern genomes except one, 9610, in which these genes were covered at only 10.91 and 11.02%, respectively. This result might be caused, however, by the 9610 genome incompleteness (the genome is at the scaffold level). The obtained results suggest that other unknown forms of these genes existed in the past or even exist today. The analysis of *potB2* and *miropsin-2* coverage in modern genomes suggests general worldwide sequence diversity. This fact is most likely reflected by the difference in protease specificity, to which inhibitor specificity had to be adjusted. In this respect, the observed variation may illustrate the evolution of inhibitors to match the changing specificity of proteases. This fact supports the thesis that a generally high Pearson correlation coefficient (0.80) was observed between the KLIKK enzyme and the corresponding POT inhibitor sequence coverage in different *T. forsythia* genomes.

Analysis of *bspA* and *bspB* revealed high sequence diversity of these genes among modern and ancient genomes. This may be related to the tandem occurrence of immunoglobulin (Ig) protein domains in BspA and BspB. The Ig-like domains share the tertiary structure despite very low conservation of the amino acid sequence. In prokaryotes they are often found on the cell surface responsible for host adhesion and presentation of ligand binding domains [46]. We speculate that *bspA* diversity is an adaptation of different strains of *T. forsythia* to specific microflora*.* PCA0332 and G12 had *bspA* sequences most similar to those presented in the modern genomes 3313 and 9610, respectively. Our findings also suggest that the Roman Iron Age and medieval *T. forsythia* (PCA0088 and PCA0198) might lack ancient or contemporary forms of *bspA*. The deletion analysis further supports our claim that the absence of *bspA* cannot be explained by random DNA degradation. As several important functions for bacterial survival have been shown for the BspA protein [6], it seems possible that an unknown homolog exerted BspA functions or that at least some of them exist in PCA0332 and in G12. This hypothesis is supported by the observation that nucleotide coverage of the *bspB* in these genomes is higher than the average coverage (that for PCA0088 was 35.56 and for PCA0198 was 30.56, whereas average nucleotide coverage was 7.03 and 9.81, respectively). *bspB* displays very high sequence similarity to *bspA*, but the former protein has not yet been investigated for its function(s). This finding may indicate that in the absence of the typical *bspA*, the mapper assigned reads to the most similar sequence in the 92A2 reference genome, i.e., to *bspB*. This finding suggests that another homolog (or homologs) of the *bspA* containing LRRs and Ig-like motifs might be present in the ancient genomes. Alternatively, PCA0088 and PCA0198 might lack a functional form of BspA, or the BspA LRR domains with diverged sequences adopted functions different than those in other *T. forsythia* strains. To answer this intriguing question longer reads (e.g. from Nanopore sequencing) might be helpful, however, only if the genomic fragment containing the *bspA* sequence was well preserved.

In contrast to sequence variations in BspA/BspB, TfsA/TfsB and Potempins/KLIKK proteases, other virulence factors involved in carbohydrate degradation and using sialic acid as a carbon source, DPPIV, OxyR, and components of the T9SS, are nearly identical in all *T. forsythia* strains. Considering very close relationship of *T. forsythia* with the human host the strict conservation of the sialic acid catabolism and transport operon apparently represents a human-specific adaptation and it is plausible that *T. forsythia* co-evolved with humans [47]. Also, the biosynthesis pathways of O-glycans decorating surface proteins, including TfsA and TfsB, with nonulosonic acid are nearly identical in the ancient and modern strains of *T. forsythia*. However, while PCA088 and PCA0198 shared the *pse* gene cluster present in ATCC 43037 and UB20, G12 had the *leg* genes as found in 92A2, UB4, KS16 and UB22. This indicates that none of these two nonulosonic acid variants provided *T. forsythia* with the cutting-edge adaptive advantage to co-exist with humans as a member of the complex dental plaque microbiome. Considering differences in host response to *T. forsythia* expressing different variants of nonulosonic acid [48], it will be interesting to see their association with the periodontal health, other periodontal pathogens and progression of periodontitis.

Among tightly conserved proteins, Miropin deserves special emphasis. As a protease inhibitor of the serpin superfamily, it possesses an exposed reactive center loop (RCL) with the residue at the P1 position and a sequence upstream of the P1-P1′ reactive site peptide bond attacked by the targeted protease that dictates the serpin’s inhibitory specificity. Using the conserved serpin scaffold, eukaryotic multicellular organisms evolved a multitude of serpins with discrete specificity dependent on variations in the RCL sequence [49]. In this context, it is very interesting to note that not only the serpin scaffold is conserved but also the RCL is identical across ancient and present-day strains of *T. forsythia*. Notably, variations in the *miropin* sequence in PCA0088 are outside the RCL. This pattern is in stark contrast to that for two recently recognized oral taxa of *Tannerella* spp. [49], which encode at least 12 serpins varying within the RCL and thus exerting different inhibitory specificities (our unpublished analysis). Of note, these strains never cluster with *T. forsythia* and may represent new species. Taken together, these data indicate that the conservation of *miropin’s* RCL through millennia suggests an essential role for this protein in *T. forsythia* “well-being” in the crowded environment of the subgingival biofilm together with highly proteolytic *P. gingivalis* and exposure to host proteases.

## Conclusion

*T. forsythia* genomes manifest high sequence similarity, with the indication that the modern worldwide genome diversity seems to be higher than that observed in the first millennium AD in Europe. Sequencing of more ancient *T. forsythia* genomes will verify this assumption. Furthermore, we showed that some virulence-associated genes (several *pot/KLIKK* and *bspA*) vary significantly between both modern and ancient *T. forsythia*. As an extreme example, the 9610 genome seems to be lacking *forsylisin*, while PCA0088 and PCA0198 most likely had more than 2 copies of a *bspA-*like of unknown sequence. This fact, together with the observed periodontitis-characteristic alveolar bone lesions in the mandible and maxilla of ancient skeletons, suggests that variations in the *bspA/Pot-KLIKK* loci did not affect the virulence of the ancient dysbiotic biofilm. Finally, we observed that the sequences of *bspA* vary significantly among modern genomes. These findings are important for further *T. forsythia* evolution studies, especially in the context of its virulence factors and adaptation to the host organism as a result of changing diet and hygienic habits.

## Methods

### Sample source

The biological material came from archaeological sites distributed across Poland. In total we examined 344 ancient human teeth, including 161 Roman Iron Age and medieval individuals from our previous studies, detailed description is available in [28, 50, 51] and 183 medieval individuals from Ląd [37], Obłaczkowo [10], Brzeg [2], Gołuń [6], Poznań–Śródka [12], Opole [41], Końskie [9], Płońsk [20], Ostrów Lednicki [20], Dziekanowice [21], and Warszawa [5] (data to be published).

### Experimental procedures

DNA was extracted from teeth in a sterile laboratory dedicated exclusively to ancient DNA (aDNA) study at the Adam Mickiewicz University in Poznan. In this laboratory, analyses on *Tannerella* cultures have never been carried out. During every step of aDNA analyses, all precautions against modern DNA contamination were kept, as previously described in [28]. At every stage of our analyses, including DNA extraction, library construction and PCR amplification (12 cycles), we set up two blanks to control potential DNA contamination. The blank controls did not yield any contaminating DNA. DNA was purified using the MinElute kit (QIAGEN) according to Yang et al. [52] and Malmstrom et al. [53]. 20 μl of DNA isolate was used to prepare a genomic library as described in Meyer and Kircher [54]. Sequencing of genomic libraries was performed with GAIIx and HiSeq 4000 (Illumina) following the standard 100 bp pair-end sequencing protocol. The average library fragment was 253 nt, 264 nt and 239 nt long for PCA0088, PCA0088 and PCA0332, respectively.

### Bioinformatics procedures

Metagenomics analyses were conducted with the default settings with MetaPhlAn2 [29], software that identifies bacteria, archaea, viruses and protozoa based on the reads mapping to marker sequences and estimates their percentage abundance in a sample. The genus habitat, Gram stain type and respiratory type were assigned manually based on the characteristics of species identified in the sample within this genus. For graphical representation, KRONA software [55] was used.

#### *T. forsythia* genome analyses

All reads were adapter-trimmed and, quality (Q ≥ 20) and length (≥20) filtered with AdapterRemoval [56]. The PCR duplicates (PCA0088 28.13% of reads, PCA0198 16.27% of reads, PCA0332 19.38% of reads and G12 19.17% of reads) were removed with picard-tools MarkDuplicates. Overlapping pair-end reads were collapsed with AdapterRemoval (−collapse option). Obtained reads were mapped to the reference *T. forsythia* genome (NC_016610.1) with bwa ver. 0.7.10 and aln -l 1000 -n 0.001 parameters following a protocol described in [57]. Subsequently, mapped files with collapsed, remaining pair-end reads and singletons were merged into one file with samtools, samtools merge [58]. Before mapping, we additionally trimmed 3 nucleotides from the beginning and 3 nucleotides from the end of the reads [57]. This step ensured that age-related transitions (C > T and G > A), which are mostly present in the last 3 bases, did not influence mapping quality and reduced SNP false positives rate. A genome coverage plot was generated with Qualimap v2.2.1 [59] based on the final mapping file (collapsed, pair-end reads and singletons). Read length distribution was assessed with PRINSEQ [60]. The consensus sequence was obtained with the command: “samtools mpileup -uf reference_genome.fna mapped.bam | bcftools view -s <(echo mapped.bam 1) -cg - | vcfutils.pl vcf2fq”. Samtools mpileup produces “pileup” textual format from an alignment file (mapped.bam). Subsequently the file was processed with bcftools view (options -cg) that outputted gVCF blocks of homozygous reference calls, with depth ranges specified and minimum allele count of sites. Finally, the consensus sequence was extracted using vcfutils.pl. The DNA damage pattern of reads (full length) that mapped to the *T. forsythia* reference genome was assessed with MapDamage2.0 [35] software with default settings.

SNP calling was performed with GATK tools [37]. Only SNPs that fulfilled the following criteria were used: (i) genotyping quality Q > 30, (ii) > 3 (or > 10) reads supported the SNP site, and (iii) the proportion of reads supporting the SNP was > 90%. If the same three criteria were fulfilled but for the reference nucleotide, we called the reference nucleotide. If neither a reference nor a SNP was called, we assigned “n” as an indicator of missing data.

#### Modern *T. forsythia* genotyping

Ten publicly available *T. forsythia* modern genomes were at different levels of assembly (3 complete genomes, 5 scaffolds, 2 contigs). To make the genotyping possible and the genomic coordinates comparable with each other, we (i) generated reads of length 100 for each of the genomes, with a shift of 1 nt using our in-home script, (ii) mapped (with bwa aln -l 1000 -n 0.001) the artificially generated reads against the reference genome (92A2), and (iii) and used on the mapping output (.bam) the same approach for SNP/reference nucleotide calling as for ancient samples (see above).

Deletions were identified based on consensus sequence analyses. Our in-home script was used to scan consensus sequences for missing blocks of DNA sequence (“n”). The full list of identified deletions is in Supplementary Table 3. Comparison of the ancient and modern genomes with the reference 92A2 was performed with CGView [42].

SNP phylogenetic tree construction from modern genomes was conducted with FastTree [38] using neighbor-joining and generalized time-reversible models of nucleotide evolution. Pplacer software [39] with the default settings was used to project the ancient stains (PCA0088, PCA0198, PCA0332, and G12) on the generated tree.

#### *BspA* analyses

The sequences of all BspA genes were retrieved from all *T. forsythia* publicly available modern genomes. We used the BLAST search tool blastn [61] and searched against the known *bspA* sequence identified in *T. forsythia* ATCC 43037. In this way, we identified the following functional *bspA* genes: BFO_RS14480 (92A2), Tanf_RS13865 (ATCC 43037), BGK60_RS08080 (9610), TF3313_RS08530 (3313), TFKS16_RS08260 (KS16), TFKS16_RS08255 (KS16), BJU00_RS03515 (UB4), BJT84_RS04075 (UB20), CLI86_11330 (NSLJ), CLI86_13580 (NSLJ) and CLI85_12020 (NSLK).

## Supplementary information

**Additional file 1.** Supplementaty Tables.

**Additional file 2.** Supplementary Material “Anthropological description of analyzed individuals” and Supplementary Figures.

## Data Availability

The datasets supporting the conclusions of this article are available in the National Center for Biotechnology Information (NCBI) Sequence Read Archive (SRA) repository under accession number: SRP093814 (https://trace.ncbi.nlm.nih.gov/Traces/sra/?study=SRP093814), and from the corresponding author on reasonable request. Data for G12 sample was downloaded from SRA (SRP029257, https://trace.ncbi.nlm.nih.gov/Traces/sra/?study=SRP029257). *T. forsythia* modern genomes were downloaded from NCBI Genome repository, 92A2 (assembly ID: GCA_000238215.1, https://www.ncbi.nlm.nih.gov/genome/11045?genome_assembly_id=231734), ATCC 43037 (assembly ID: GCA_006385365.1, https://www.ncbi.nlm.nih.gov/genome/11045?genome_assembly_id=590153), 9610 (assembly ID: GCA_001938785.1, https://www.ncbi.nlm.nih.gov/genome/11045?genome_assembly_id=300815), 3313 (assembly ID: GCA_001547875.1, https://www.ncbi.nlm.nih.gov/genome/11045?genome_assembly_id=264125), KS16 (assembly ID: GCA_001547855.1, https://www.ncbi.nlm.nih.gov/genome/11045?genome_assembly_id=264124), UB4 (assembly ID: GCA_900096725.1, https://www.ncbi.nlm.nih.gov/genome/11045?genome_assembly_id=284387), UB20 (assembly ID: GCA_900096735.1, https://www.ncbi.nlm.nih.gov/genome/11045?genome_assembly_id=284388), UB22 (assembly ID: GCA_900096715.1, https://www.ncbi.nlm.nih.gov/genome/11045?genome_assembly_id=284386), NSLJ (assembly ID: GCA_002529085.1, https://www.ncbi.nlm.nih.gov/genome/11045?genome_assembly_id=340904), NSLK (assembly ID: GCA_002529295.1, https://www.ncbi.nlm.nih.gov/genome/11045?genome_assembly_id=340905). *bspA* sequence of *T. forsythia* ATCC 43037 was obtained from NCBI Gene repository (locus tag: BFO_RS14480, https://www.ncbi.nlm.nih.gov/gene/34760141). The *bspA* sequences for the remaining modern *T. forsythia* were obtained by blastn search of their genomes (against BFO_RS14480). As a result we identified the following *bspA-like* genes BFO_RS14480 (92A2), Tanf_RS13865 (ATCC 43037), BGK60_RS08080 (9610), TF3313_RS08530 (3313), TFKS16_RS08260 (KS16), TFKS16_RS08255 (KS16), BJU00_RS03515 (UB4), BJT84_RS04075 (UB20), CLI86_11330 (NSLJ), CLI86_13580 (NSLJ) and CLI85_12020 (NSLK) which are available in NCBI Nucleotide repository. KLIKK *locus* sequence was obtained from NCBI Nucleotide repository (accession IDs: KP715368 https://www.ncbi.nlm.nih.gov/nuccore/KP715368 and KP715369 https://www.ncbi.nlm.nih.gov/nuccore/KP715369).

## Abbreviations

aDNA: Ancient DNA
NGS: Next Generation Sequencing
LRR: Leucine-rich repeat
Ig-like: Immunoglobulin-like
SNP: Single nucleotide polymorphism
Ig: Immunoglobulin
RCL: Reactive center loop
