# Supplementary material for: Analysis of oral microbiome from fossil human remains revealed the significant differences in virulence factors of modern and ancient Tannerella forsythia
Source: BMC Genomics. 2020 Jun 15;21:402. doi: 10.1186/s12864-020-06810-9 (PMC7296668; doi:10.1186/s12864-020-06810-9)
Supplement: Supplementary file 2 — Additional file 2. Supplementary Material “Anthropological description of analyzed individuals” and Supplementary Figures. [file 12864_2020_6810_MOESM2_ESM.pdf]

SUPPLEMENTARY MATERIAL

Anthropological description of analyzed individuals

The three individuals, from which we obtained sufficient amount of *T. forsythia* DNA sequences (PCA0088, PCA0198 and PCA0332), were analyzed morphologically in order to determine their sex and age, as well as to trace possible signs of periodontitis caused by oral pathogens.

Sample PCA0088 belonged to 15 ± 3 year old individual which was excavated from the roman-age cemetery in Masłomęcz, dated from the 2nd to the 4th c. AD (1-3). The sex of examined individual was not determined due to its juvenile age at death. The state of preservation of the cranium was poor and only particular, but not complete, bones remained (e.g. the left temporal bone and the occipital bone). Most parts of the facial skeleton were lacking, except the left part of the mandible and a nearly complete left maxilla with fragment of the zygomatic bone. The assessment of the surface and texture of the interdental septal alveolar bone indicated the presence of moderate periodontitis in the maxilla and mandible in the alveolar bone, both, between upper P4 and M1, and lower M1 and M2 (Supplementary Figure 2 A). Due to poor preservation of human nuclear DNA, the molecular sex of this individual was not obtained, however U3a1a mitochondrial haplogroup, typical for north Europe, was identified.

Sample PCA0198 belonged to the 45-55 year old man who was excavated form the early medieval necropolis in Ląd archeological site in Poland. The necropolis was dated to the period from the second half of the 10th to the $11^{th}/12^{th}$ c. AD. The bones of the skeleton were well preserved. Complete cranium with slightly damaged interiors of the eye sockets and base of the skull, showed traces of inflammation of the alveolar process of the maxillas (Supplementary Figure 2 B). Ancient DNA analyses conducted for this individual confirmed his male sex. Collagen sample from PCA0198 was AMS 14C-dated at the Poznań Radiocarbon Laboratory, in Poland. Analyzed individual was dated to 1117AD-1225AD with 95.40% probability (Supplementary Figure 3) what confirmed its medieval origin.

Individual PCA0332 was a 35-45 year old man, excavated form medieval necropolis in Ostrów Lednicki, in Poland. This individual was well preserved with complete skull and postcranial skeleton. Both, mandible and maxilla had clear signs of chronic periodontitis and dental abscess (Supplementary Figure 2 C). Additionally, in mandible periapical lesions were found in the

form of traces of root molar abscesses. Male sex determined by anthropological analyses was confirmed by the analysis of aDNA. The mitochondrial and Y DNA haplogroups were not possible to determine due to not sufficient SNPs coverage.

Supplementary materials and methods

Human mitochondrial and nuclear genome analyses. Read mapping was performed with a BWA software (4) using hg19 as a reference as described in (5). SNP calling was performed with Freebayes (6). Mitochondrial DNA haplogroups were assigned with (7).

Supplementary references

1. Kokowski A. Grupa masłomęcka i jej cmentarzyska w: Starożytna Polska. Od trzeciego wieku przed Chrystusem do starożytności. Wydawnictwo TRIO. 2006;1:385–412.
2. Kokowski A. Archeologia Gotów. Goci z Kotliny Hrubieszowskiej. Idea Media. 1999.
3. Stolarek I, Handschuh L, Juras A, Nowaczewska W, Kocka-Krenz H, Michalowski A, et al. Goth migration induced changes in the matrilineal genetic structure of the central-east European population. Sci Rep. 2019;9(1):6737.
4. Li H, Durbin R. Fast and accurate short read alignment with Burrows-Wheeler transform. Bioinformatics. 2009;25(14):1754-60.
5. Schubert M, Ginolhac A, Lindgreen S, Thompson JF, Al-Rasheid KA, Willerslev E, et al. Improving ancient DNA read mapping against modern reference genomes. BMC Genomics. 2012;13:178.
6. Garrison E, Marth G. Haplotype-based variant detection from short-read sequencing. arXivorg. 2012; arXiv:1207.3907v2 ([q-bio.GN]).
7. Vianello D, Sevini F, Castellani G, Lomartire L, Capri M, Franceschi C. HAPLOFIND: a new method for high-throughput mtDNA haplogroup assignment. Human mutation. 2013;34(9):1189-94.

SUPPLEMENTARY FIGURES

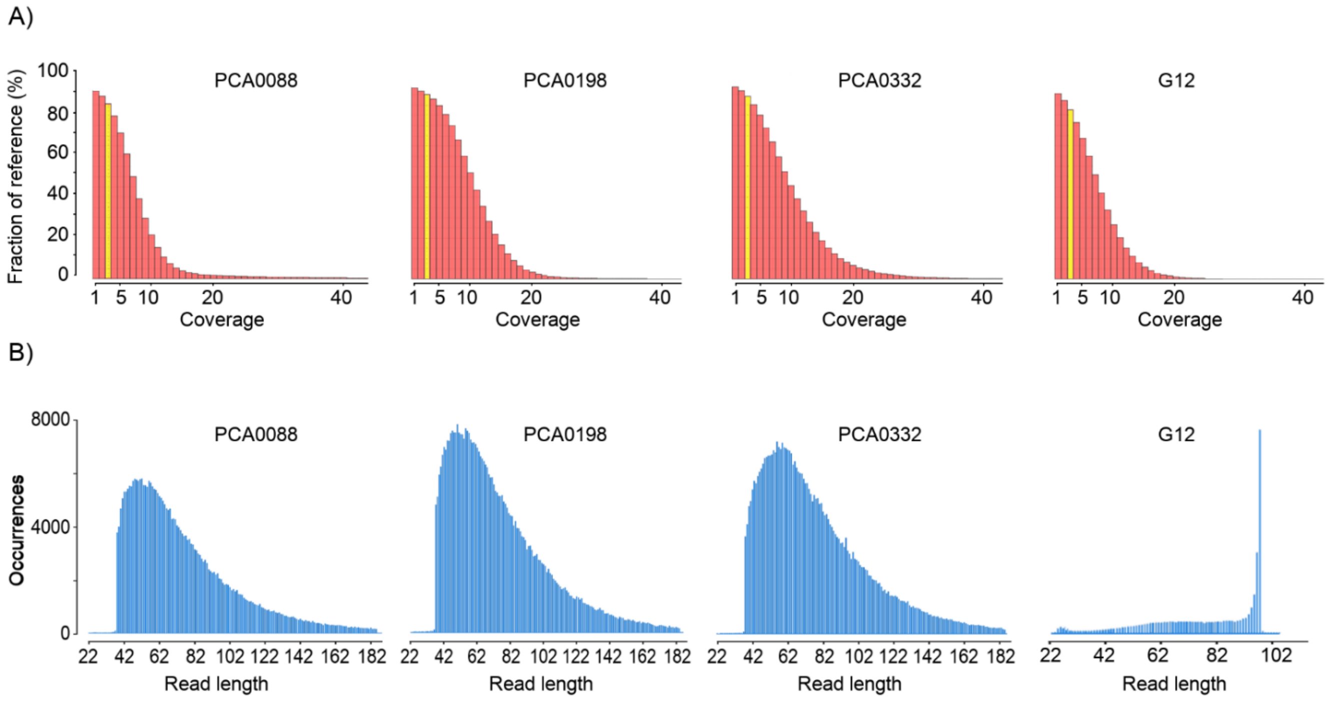

Supplementary Figure 1. A) the percentage of *T. forsythia* genome bases coverage; B) The distribution of merged reads (PCA0088, PCA0198, PCA0332) and single-end reads (G12) length that mapped to the *T. forsythia* reference genome (NC_016610.1). Reads < 20 nt. were excluded in the filtration process as non-informative.

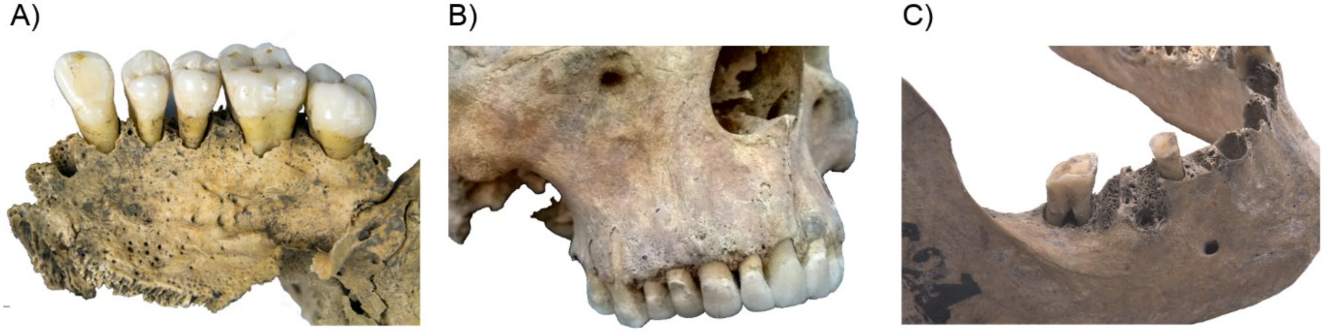

Supplementary Figure 2. The jaw remains with visible inflammatory lesion of individuals form A) PCA0088, Masłomęcz; B) PCA0198, Ląd; C) PCA0332, Ostrów Lednicki.

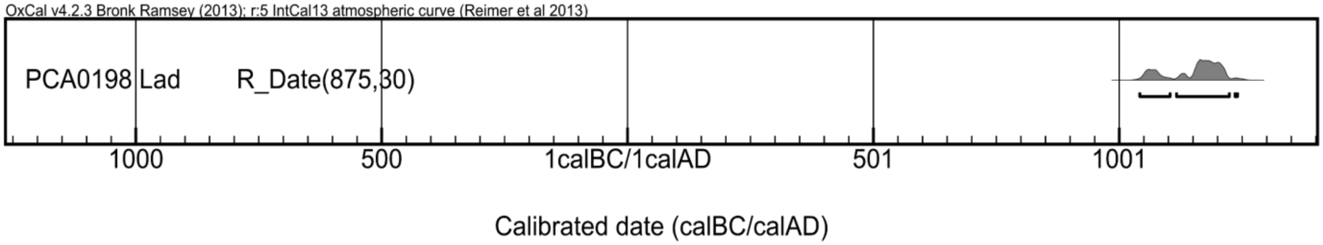

Supplementary Figure 3. The radiocarbon dating of medieval sample PCA0198.

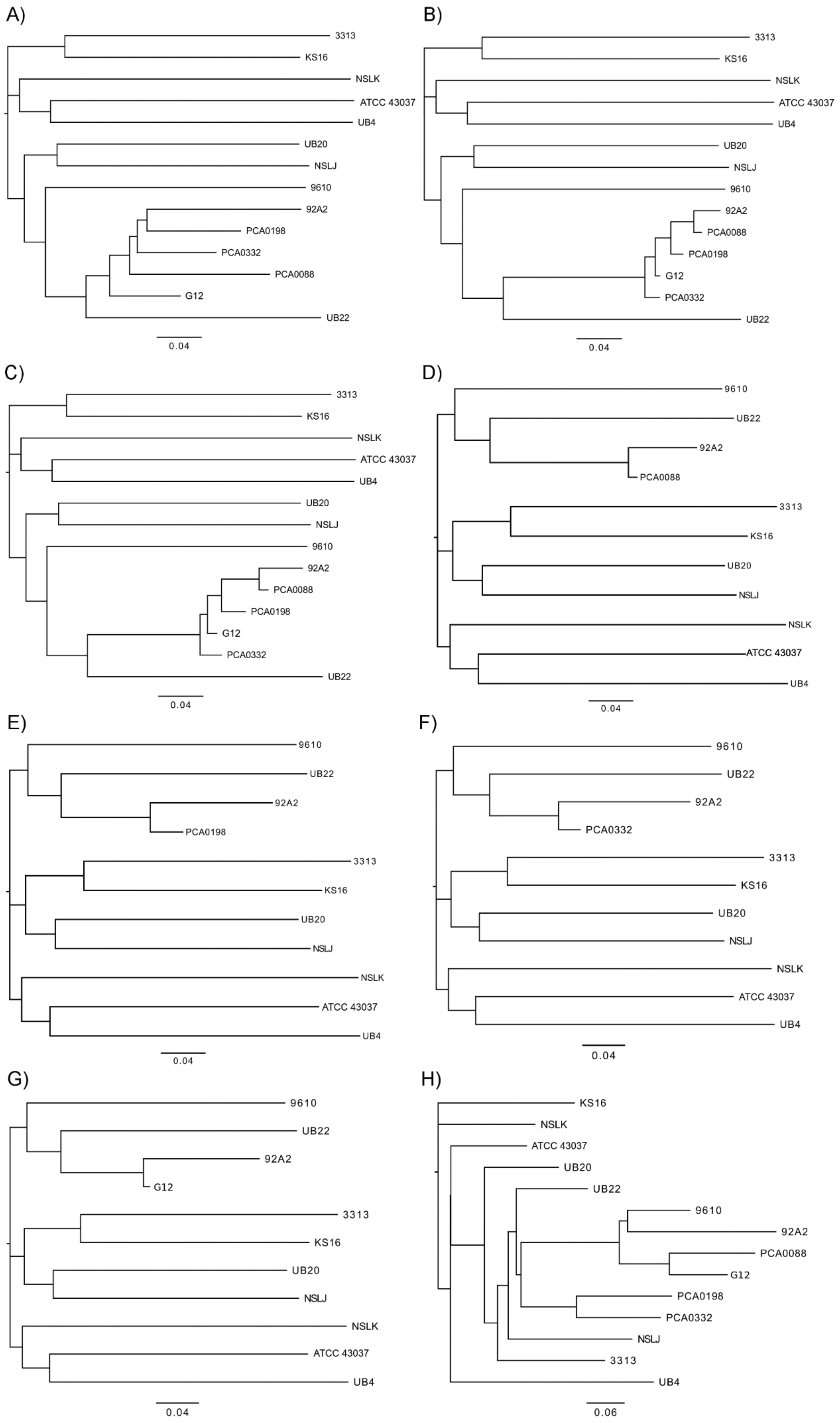
A)
3313
KS16
NSLK
ATCC 43037
UB4
UB20
NSLJ
9610
92A2
PCA0198
PCA0332
PCA0088
G12
UB22
0.04
B)
3313
KS16
NSLK
ATCC 43037
UB4
UB20
NSLJ
9610
92A2
PCA0088
PCA0198
G12
PCA0332
UB22
0.04
C)
3313
KS16
NSLK
ATCC 43037
UB4
UB20
NSLJ
9610
92A2
PCA0088
PCA0198
G12
PCA0332
UB22
0.04
D)
9610
UB22
92A2
PCA0088
3313
KS16
UB20
NSLJ
NSLK
ATCC 43037
UB4
0.04
E)
9610
UB22
92A2
PCA0198
3313
KS16
UB20
NSLJ
NSLK
ATCC 43037
UB4
0.04
F)
9610
UB22
92A2
PCA0332
3313
KS16
UB20
NSLJ
NSLK
ATCC 43037
UB4
0.04
G)
9610
UB22
92A2
G12
3313
KS16
UB20
NSLJ
NSLK
ATCC 43037
UB4
0.04
H)
KS16
NSLK
ATCC 43037
UB20
UB22
9610
92A2
PCA0088
G12
PCA0198
PCA0332
NSLJ
3313
UB4
0.06

Supplementary Figure 4. SNP phylogenetic trees showing the positions of roman-age PCA0088 and medieval PCA0198, PCA0332, G12 *T. forsythia* genomes in a respect to the modern worldwide occurring *T. forsythia*. A) SNP/reference base was called if at least 10-fold coverage was observed at the position. B) Reads <70 nt. long were excluded C) SNPs C/T and G/A were excluded. D) SNP tree was calculated for modern and PCA0088 genomes. E) SNP tree was calculated for modern and PCA0198 genomes. F) SNP tree was calculated for modern and PCA0332 genomes. G) SNP tree was calculated for modern and G12 genomes. H) SNP/reference base was called if at least 3-fold coverage was observed at the position and ATCC 43037 genome was used as a reference.

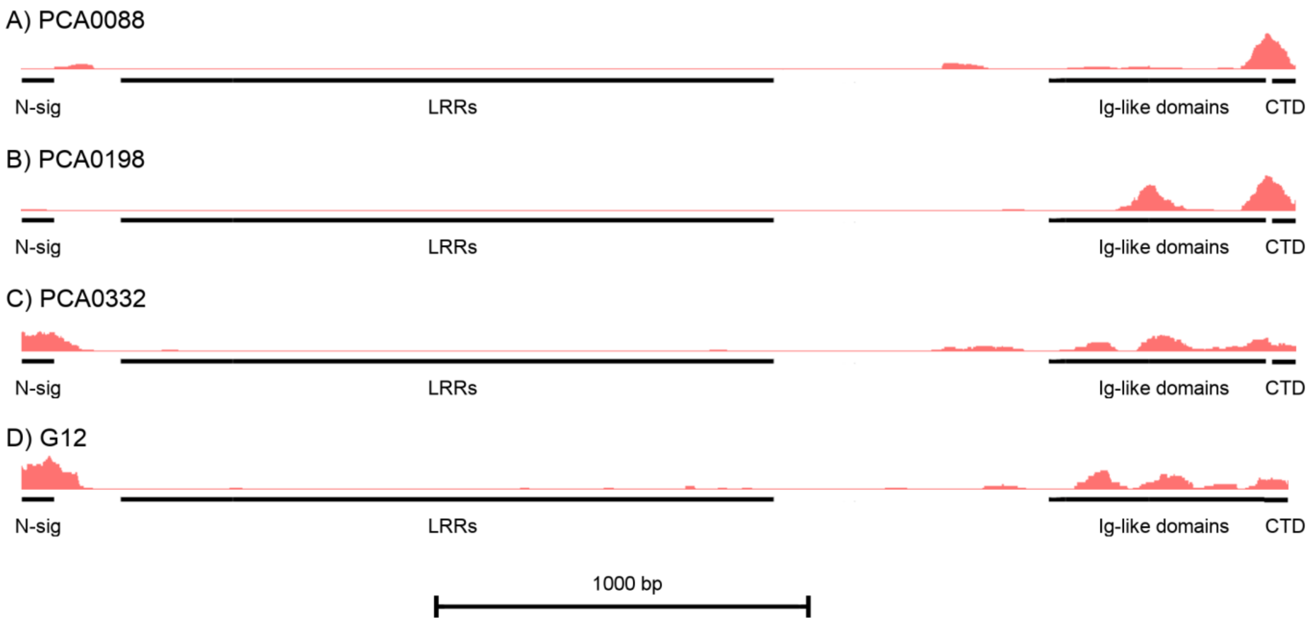

Supplementary Figure 5. Schematic representation of *bspA* and its coverage in A) PCA0088, B) PCA0198, C) PCA0332, D) G12.

A)

	UB22	UB20	ATCC 43037	9610	UB4	92A2	3313	NSLK	KS16	NSLJ
TF2998		1	1	1	1	1	1	1	2	2
TF1843			1	1	1	1	1	1	1	
TF2922	1	1		1	1	1	1	1	2	1
TF0535	1	1	1		1	1	1	1	1	1
TF1591	1	1	1	1		1	1	1		1
TF1589		1	1	2	2	1	1	2	2	1

B)

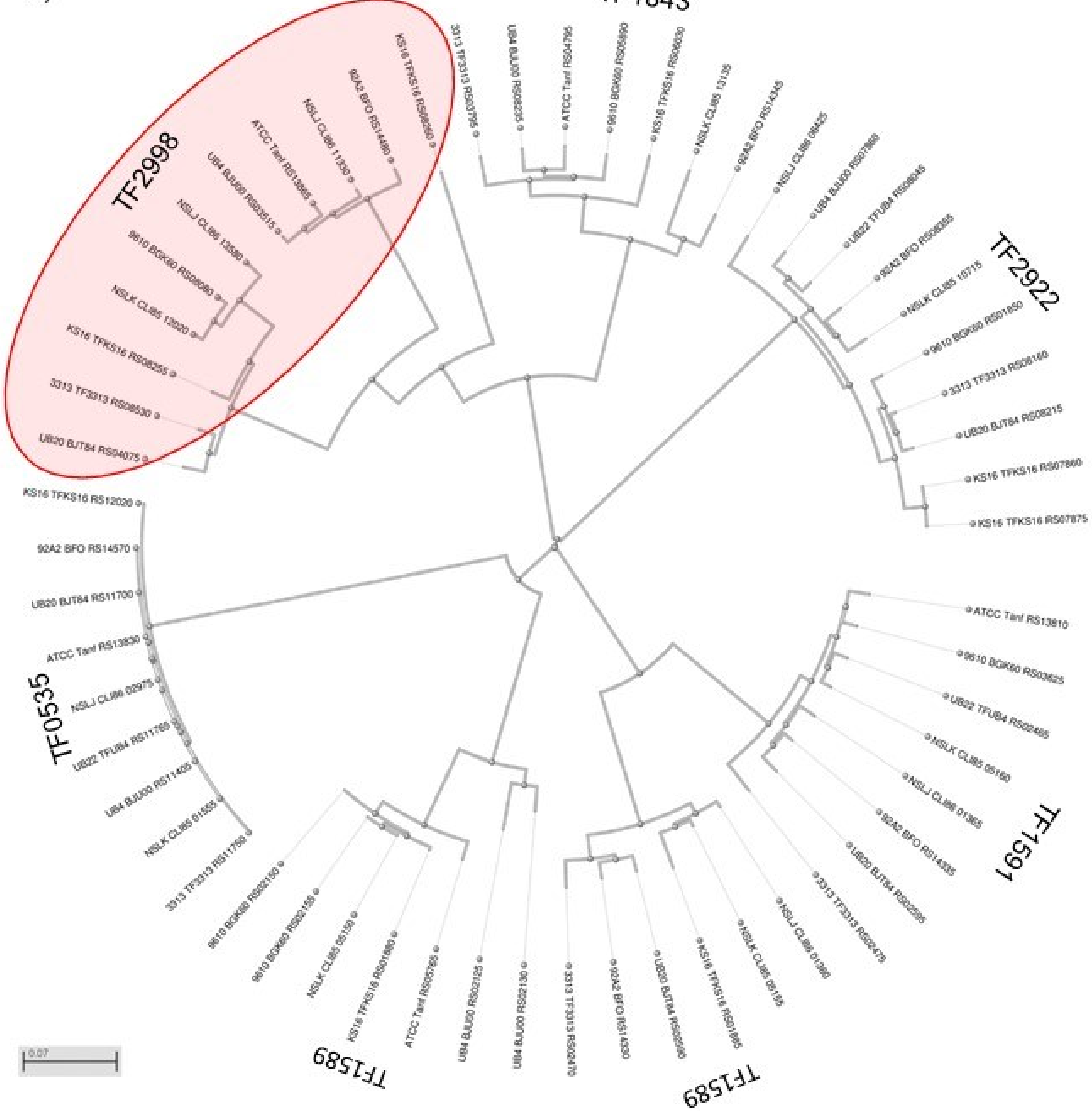

Supplementary Figure 6. A) the list of *T. forsythia bspA* homologues identified in the modern strains. B) The phylogenetic thee of *bspA* homologues. The functional *bspAs* are highlighted in red.

A)

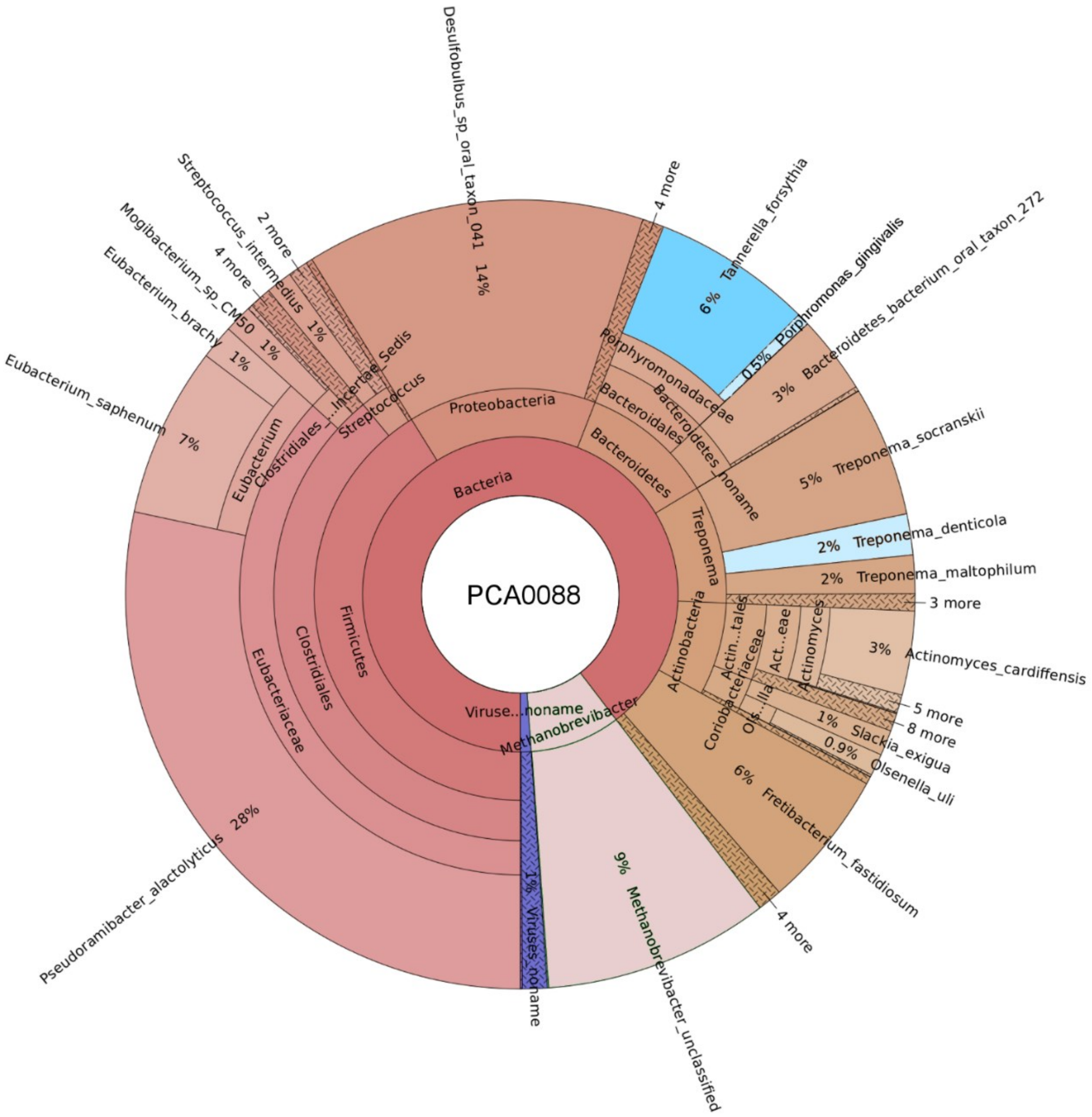

Respiratory type [%]		Gram-stain type [%]		Habitat [%]	
Aerobic	67.27	Gram+	47.76	Oral	75.34
Facultative aerobic	8.16	Gram-	51.66	Other-human	7.48
Anaerobic	67.27			Pathogen	2.26
Facultative anaerobic	8.16			Environment	14.35

B)

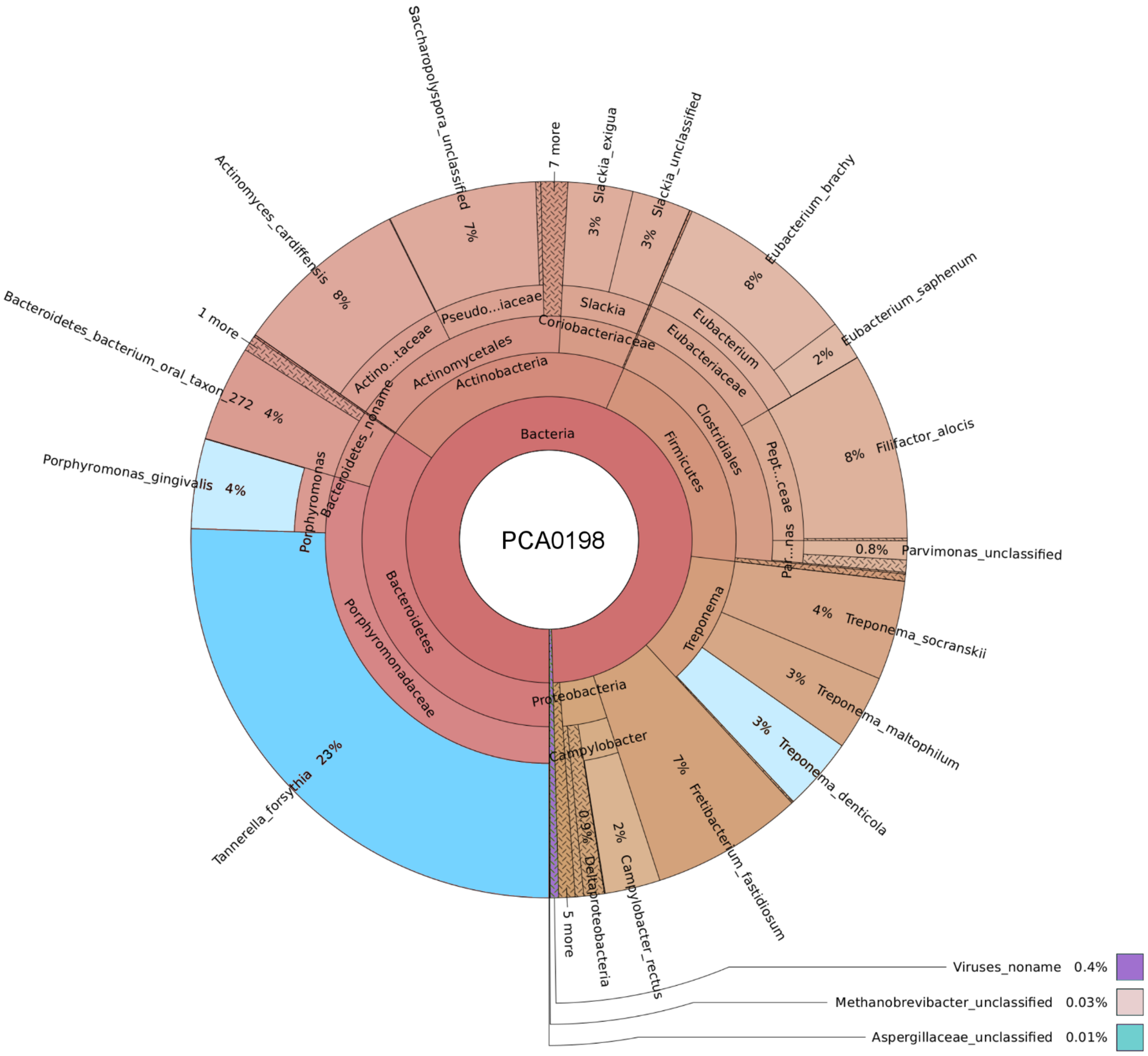

Respiratory type [%]		Gram-stain type [%]		Habitat [%]	
Aerobic	2.92	Gram+	60.29	Oral	77.63
Facultative aerobic	0.55	Gram-	37.61	Other-human	17.68
Anaerobic	88.60			Pathogen	0.91
Facultative anaerobic	5.82			Environment	1.93

C)

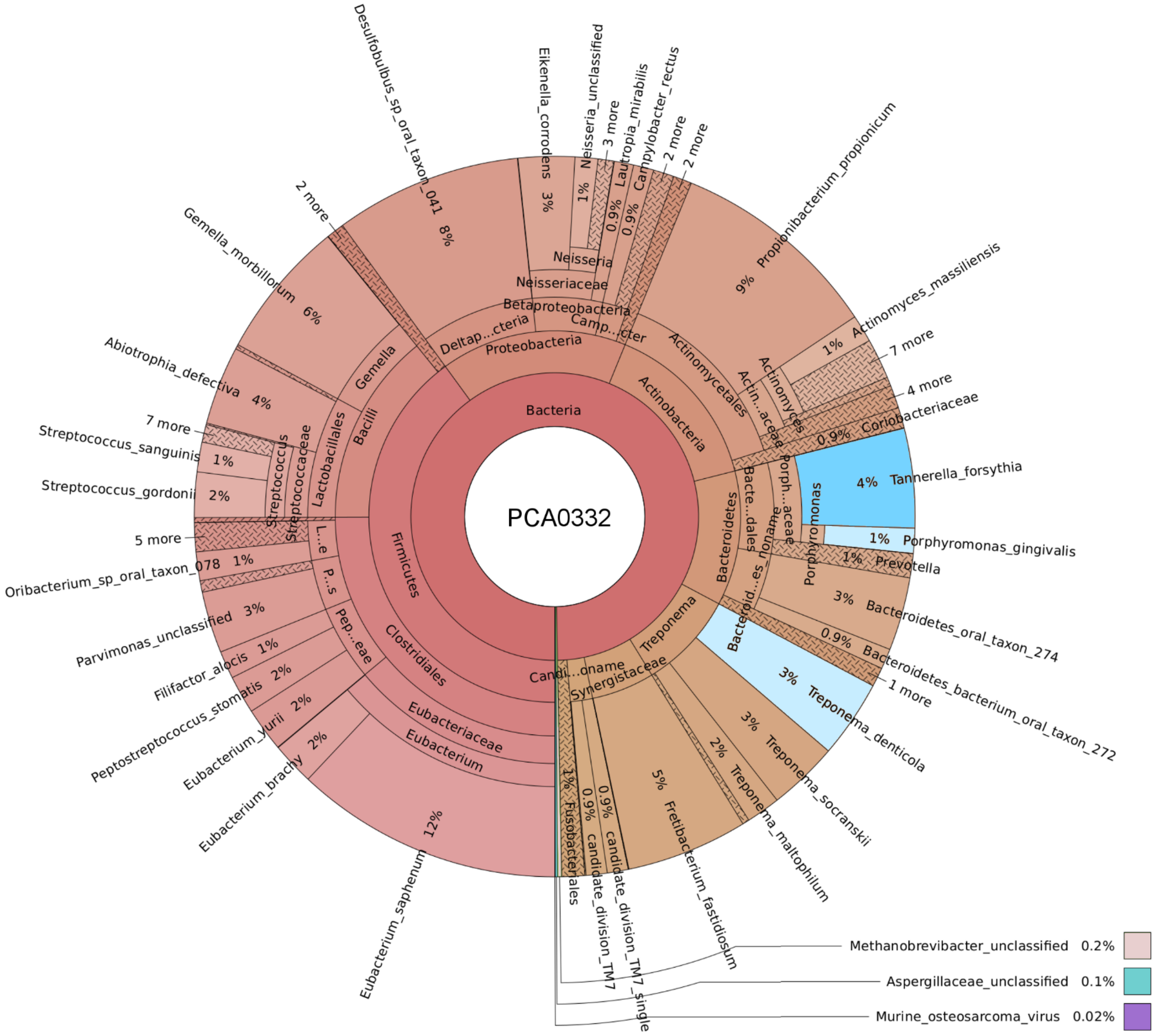

Respiratory type [%]		Gram-stain type [%]		Habitat [%]	
Aerobic	11.90	Gram+	52.71	Oral	90.42
Facultative aerobic	5.09	Gram-	45.22	Other-human	5.31
Anaerobic	60.36			Pathogen	1.75
Facultative anaerobic	20.59			Environment	2.28

D)

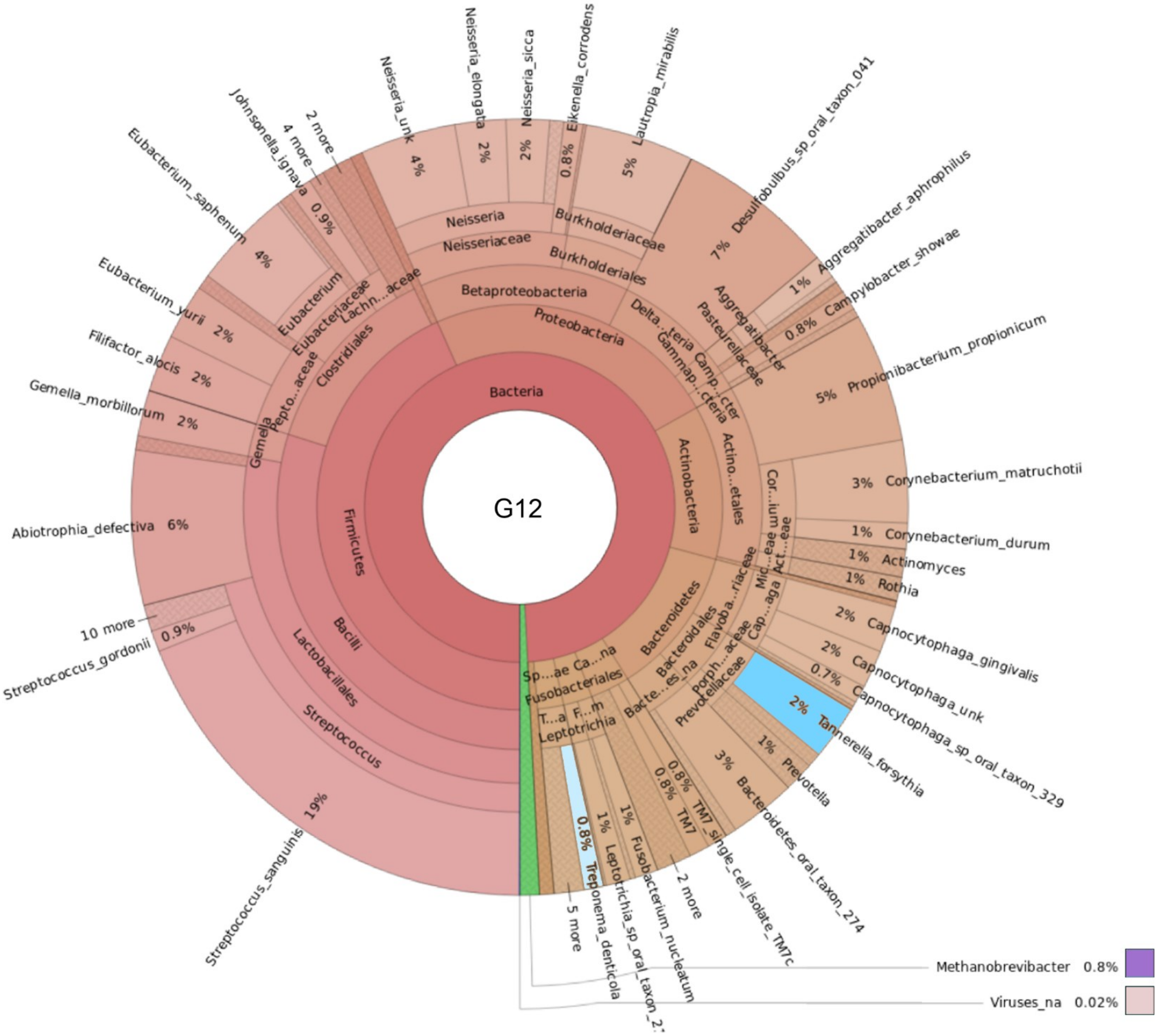

Respiratory type [%]		Gram-stain type [%]		Habitat [%]	
Aerobic	23.77	Gram+	50.06	Oral	81.04
Facultative aerobic	7.16	Gram-	46.97	Other-human	13.87
Anaerobic	31.70			Pathogen	1.13
Facultative anaerobic	34.41			Environment	3.94

Supplementary Figure 7. Taxonomic overview of the sequence reads in A) PCA0088 B) PCA0198 C) PCA0332 D) G12 shotgun dataset. The fractions of reads assigned to *T. forsythia* are in blue. The fractions of *P. gingivalis* and *T. denticola* reads are displayed in light blue.
